# The Use of Tau PET to Stage Alzheimer Disease According to the Braak Staging Framework

**DOI:** 10.2967/jnumed.122.265200

**Published:** 2023-08

**Authors:** Arthur C. Macedo, Cécile Tissot, Joseph Therriault, Stijn Servaes, Yi-Ting Wang, Jaime Fernandez-Arias, Nesrine Rahmouni, Firoza Z. Lussier, Marie Vermeiren, Gleb Bezgin, Paolo Vitali, Kok Pin Ng, Eduardo R. Zimmer, Marie-Christine Guiot, Tharick A. Pascoal, Serge Gauthier, Pedro Rosa-Neto

**Affiliations:** 1 Department of Neurology and Neurosurgery, McGill University, Montréal, Québec, Canada;; 2 Department of Psychiatry and Neurology, University of Pittsburgh, Pittsburgh, Pennsylvania;; 3 Department of Neurology, National Neuroscience Institute, Singapore, Singapore;; 4 Department of Pharmacology, Universidade Federal do Rio Grande do Sul, Porto Alegre, Brazil; and; 5 Department of Pathology, McGill Hospital Center, Montréal, Québec, Canada

**Keywords:** Alzheimer disease, Braak staging, PET, neurofibrillary tangles, cognitive impairment

## Abstract

Amyloid-β plaques and neurofibrillary tangles (NFTs) are the 2 histopathologic hallmarks of Alzheimer disease (AD). On the basis of the pattern of NFT distribution in the brain, Braak and Braak proposed a histopathologic staging system for AD. Braak staging provides a compelling framework for staging and monitoring of NFT progression in vivo using PET imaging. Because AD staging remains based on clinical features, there is an unmet need to translate neuropathologic staging to a biologic clinical staging system. Such a biomarker staging system might play a role in staging preclinical AD or in improving recruitment strategies for clinical trials. Here, we review the literature regarding AD staging with the Braak framework using tau PET imaging, here called PET-based Braak staging. Our aim is to summarize the efforts of implementing Braak staging using PET and assess correspondence with the Braak histopathologic descriptions and with AD biomarkers. **Methods:** We conducted a systematic literature search in May 2022 on PubMed and Scopus combining the terms “Alzheimer” AND “Braak” AND (“positron emission tomography” OR “PET”). **Results:** The database search returned 262 results, and after assessment for eligibility, 21 studies were selected. Overall, most studies indicate that PET-based Braak staging may be an efficient method to stage AD since it presents an adequate ability to discriminate between phases of the AD continuum and correlates with clinical, fluid, and imaging biomarkers of AD. However, the translation of the original Braak descriptions to tau PET was done taking into account the limitations of this imaging technique. This led to important interstudy variability in the anatomic definitions of Braak stage regions of interest. **Conclusion:** Refinements in this staging system are necessary to incorporate atypical variants and Braak-nonconformant cases. Further studies are needed to understand the possible applications of PET-based Braak staging to clinical practice and research. Furthermore, there is a need for standardization in the topographic definitions of Braak stage regions of interest to guarantee reproducibility and methodologic homogeneity across studies.

Alzheimer disease (AD) is a progressive neurodegenerative disease whose neuropathologic hallmarks are amyloid-β (Aβ) plaques and neurofibrillary changes (1). Three types of neurofibrillary changes underlie AD pathogenesis: neuritic plaques, neurofibrillary tangles (NFTs), and neuropil threads. NFT and neuropil thread accumulation in the cerebral cortex present a well-defined distribution and progression pattern, allowing the differentiation of AD into stages (2–5). On this basis, Braak and Braak proposed a neuropathologic staging system comprising 6 successive stages (6). In 2006, this framework was revised, incorporating modern immunohistochemical techniques to improve its applicability and accuracy (7).

Braak stages are hierarchic, meaning that a given stage encompasses the abnormalities observed in the earlier ones (6*,*^7^). Stages I and II are marked by the involvement of the transentorhinal and entorhinal cortices, respectively (7). At stage III, modest damage is seen in the hippocampus, amygdala, and adjacent neocortical areas (7). Stage IV defines the initial extension of the pathologic process to neocortical association areas, encompassing the insular cortex and basal frontal areas (7). Stage V shows an impairment of neocortical association areas, especially in temporal, parietal, and associative occipital regions (7). In stage VI, degeneration spreads to primary motor and sensory fields. For practical purposes, these stages may be simplified into 3 categories: transentorhinal (I–II), limbic (III–IV), and isocortical (V–VI) (8). In 2012, this staging system was integrated into the AD neuropathologic diagnostic criteria (9*,*^10^). Figure 1 displays the topography of Braak stages according to the original histopathologic descriptions.

**FIGURE 1.**
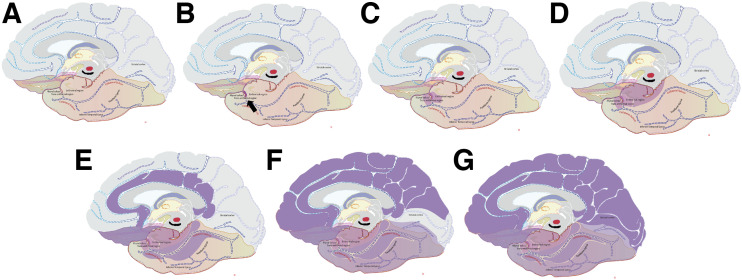
Topographic representation of Braak stages 0 (absence of tau accumulation) (A), I (B), II (C), III (D), IV (E), V (F), and VI (G) according to original histopathologic descriptions, displaying sagittal section of brain in midline. Affected brain regions are colored in different shades of purple.

Because AD staging remains based on clinical features, there is an unmet need to translate neuropathologic staging to a biologic clinical staging system. Such a biomarker staging system might play a role in diagnosing preclinical AD and in improving recruitment strategies for clinical trials. The development of tau PET ligands created a way to map tau accumulation in the brain of living humans over time and stratify individuals on the AD continuum on the basis of in vivo Braak staging (11). By contrast, pathology remains limited in detecting longitudinal changes in tau accumulation, in establishing correlations to other biomarkers, and in its applicability in clinical practice and research. Compared with histopathologic Braak assessment, which is conducted in a limited number of regions, PET provides an overview of tau pathology across the entire brain. PET limitations, in turn, include poorer resolution and sensitivity than neuropathology, as well as contamination of the early stages of NFT accumulation with off-target binding, especially in first-generation tau PET agents (12). Together, these limitations impose significant variability to parallel the in vivo classification system with the Braak histopathological scheme.

The recently developed second-generation tau PET ligands (^18^F-MK6240, ^18^F-PI2620, and ^18^F-RO948) presenting higher sensitivity to tau and less off-target binding mitigated some of the limitations of first-generation agents (^18^F-AV1451 and ^18^F-THK5351) and raised expectations about the implementation of Braaklike systems using in vivo techniques (13–17). Here, we review the literature regarding AD staging with the Braak histopathologic framework using tau PET imaging, hereafter called PET-based Braak staging. Our aim is to summarize the efforts to implement Braak staging using PET and assess their correspondence with the Braak histopathologic descriptions and with AD biomarkers.

## MATERIALS AND METHODS

A literature search was conducted on May 9, 2022, on PubMed and Scopus, using the following terms: “Alzheimer” AND “Braak” AND (“positron emission tomography” OR “PET”). No restrictions regarding language, year of publication, or type of report were applied. Two authors independently screened titles, abstracts, and full texts on the basis of the following inclusion criteria: cross-sectional, cohort, or case-control studies testing the applicability of PET-based Braak staging in the AD continuum. The exclusion criteria were unpublished manuscripts, animal or in vitro studies, reviews, clinical trials, case reports or series, conference papers, editorials, letters, responses, comments, book chapters, and guidelines.

**NOTEWORTHY**

- Because AD staging remains based on clinical features, there is an unmet need to translate neuropathologic staging to a biologic clinical staging system.
- The development of tau PET ligands created a way to map tau accumulation in the brain of living humans over time and stratify individuals on the AD continuum based on in vivo Braak staging.
- Most studies indicate that tau PET performs well in staging AD when using Braak ROIs, even when compared with other biomarkers and clinical predictors.
- Refinements in the PET-based Braak staging system are necessary to incorporate atypical and Braak-nonconformant cases.
- The harmonization of Braak ROIs might represent a further step for ascertaining reproducibility.

The following data were extracted by 2 independent assessors: authors, year of publication, study design, population size and diagnoses, type of tau PET ligand used, statistical or practical approach to assessing PET-based Braak stages, main results, topographic definition of Braak stage regions of interest (ROIs), and meta-ROIs. Disagreements were resolved by consensus or by consulting a third investigator.

## RESULTS

### Study Selection

**FIGURE 2.**
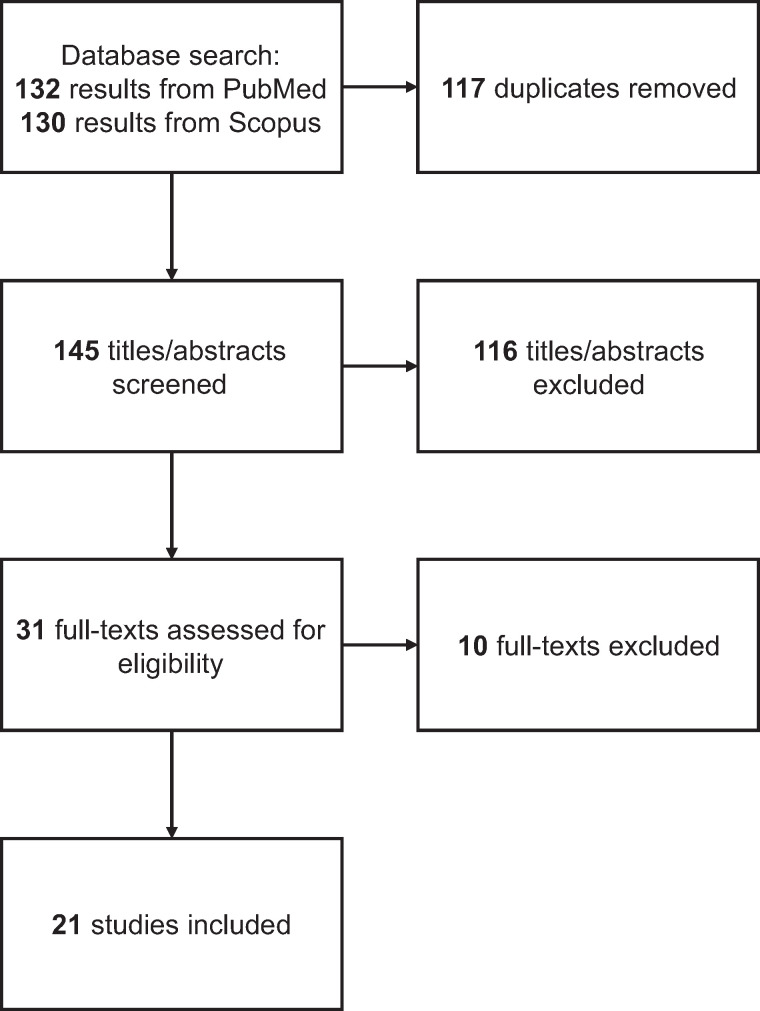
Flowchart of study selection.

The database search returned 132 and 130 results from PubMed and Scopus, respectively. After extraction of 117 duplicates, 145 records had their titles and abstracts screened for inclusion, leading to 31 reports selected for full-text evaluation. Ten reports were excluded for not assessing the use of PET-based Braak staging in the AD continuum, leaving 21 studies to be included in our review. Figure 2 displays the flowchart of study selection, and Table 1 shows the characteristics and main results of the studies included.

**TABLE 1.** Characteristics and Main Results of Included studies

Study	Design	Sample and clinical diagnoses	Tau PET ligand	Main results
Schöll (11)	Cohort	15 AD (1 fvAD, 1 amnestic EOAD, 3 amnestic LOAD, 4 lvPPA, 6 PCA), 38 CU	^18^F-AV1451	Tracer retention corresponded well with Braak staging and was related to cognitive measures
Schwarz (24)	Cross-sectional	44 AD*, 87 MCI, 56 CU	^18^F-AV1451	Binding strongly followed Braak scheme, but atypical patterns were also seen; Braak stages correlated with cognitive decline, diagnosis, and Aβ load
Lowe (36)	Cross-sectional	5 AD, 3 MCI, 7 CU, 21 non-AD	^18^F-AV1451	Binding did not completely reflect early-stage tau progression suggested by Braak in atypical AD
Maass (18)	Cross-sectional	Sample 1: 48 AD^†^, 12 MCI, 86 CU/sample 2: 9 amnestic LOAD, 19 MCI, 42 CU	^18^F-AV1451	Braak-based staging, whole-brain, and regional tau measures had similar accuracies to distinguish MCI and AD from CU individuals
Marquié (35)	Cross-sectional	22 NR	^18^F-AV1451	Autoradiographic ^18^F-AV1451 binding correlated with NFT accumulation and matched Braak stage progression
Lowe (34)	Cross-sectional	51 AD^†^, 35 MCI, 601 CU	^18^F-AV1451	Distribution of tau PET signal in CU individuals was similar but not identical to Braak descriptions
Cho (33)	Cohort	25 amnestic AD, 31 MCI, 52 CU	^18^F-AV1451	Tau accumulation supported Braak model and was associated with cognitive dysfunction
Timmers (31)	Cross-sectional	52 AD^†^, 6 MCI, 42 CU	^18^F-AV1451	^18^F-AV1451 in Braak I–II, III–IV, and V–VI was related to cortical atrophy in MCI/AD patients
Baek (32)	Cohort	29 AD*, 39 MCI, 164 CU	^18^F-AV1451	Observed sequence of tau accumulation supported spreading model proposed by Braak and Braak
Betthauser (19)	Cohort	167 CU	^18^F-MK6240	Binding mostly followed Braak stages and seemed to be disease-dependent
Franzmeier (20)	Cross-sectional	11 amnestic AD, 85 MCI, 348 CU	^18^F-AV1451	Tau deposition in AD did not always follow Braak scheme, as may be explained by variability in tau epicenters
Leuzy (29)	Cross-sectional	100 AD*, 154 MCI, 257 CU, 102 non-AD	^18^F-RO948	Braak ROI SUV ratio could discriminate AD from other neurodegenerative diseases, MCI, and controls
Pascoal (17)	Cross-sectional	54 AD (30 EOAD, 21 LOAD, 3 NR), 67 MCI, 168 CU, 12 FTD	^18^F-MK6240	Ligand uptake recapitulated Braak stages, which were related to Aβ status, neurodegeneration, and cognitive impairment
Shokouhi (21)	Cross-sectional	160 MCI, 301 CU	^18^F-AV1451	Tau-based network architecture mirrored Braak stage progression
Kim (30)	Cross-sectional	26 AD*, 55 MCI, 32 CU	^18^F-THK5351	MRI brain volumetry and Braak ROI SUV ratio showed robust performance in discriminating AD spectrum
Kreisl (22)	Cross-sectional	40 AD*, 22 MCI, 39 CU	^18^F-MK6240	Binding followed Braaklike progression and correlated with amyloid positivity, CSF biomarkers, cognition, and clinical diagnosis
Pascoal (25)	Cohort	17 AD*, 21 MCI, 87 CU	^18^F-MK6240	Tau progression followed Braak stages and might be detected in vivo in individuals with and without symptoms of AD
Seemiller (28)	Cross-sectional	39 AD*, 39 CU	^18^F-AV1451	Tau distribution was consistent with Braak staging model and supported network degeneration hypothesis
Nihashi (23)	Cross-sectional	17 AD*, 9 MCI, 43 CU	^18^F-THK5351	Binding in Braak ROIs was higher in MCI and AD than in CU but did not differ significantly between MCI and AD
Rullmann (27)	Cross-sectional	37 AD*, 26 CU	^18^F-PI2620	^18^F-PI-2620 binding widely recapitulated Braak descriptions, and tau signal correlated with poorer cognition in all stages but VI
Therriault (26)	Cohort	65 AD^†^, 80 MCI, 179 CU	^18^F-MK6240	PET-based Braak staging presented stage-specific correlations with Aβ PET abnormality, CSF and plasma phosphorylated tau biomarkers, and dementia severity

* Study did not specify whether different AD variants were included.

† Study included different AD variants but did not provide number of participants with each variant.

fvAD = frontal variant AD; EOAD = early-onset AD; LOAD = late-onset AD; FTD = frontotemporal dementia; lvPPA = logopenic variant primary progressive aphasia; PCA = posterior cortical atrophy; NR = not reported.

### Variability in Definitions of PET-Based Braak Stages Across Studies

The ROIs used to define PET-based Braak stages showed some variability across studies (Supplemental Table 1; supplemental materials are available at http://jnm.snmjournals.org). Stage I was characterized as the entorhinal cortex in 7 studies (11*,*^18^–^23^) and as the transentorhinal cortex in 6 studies (17*,*^24^–^27^). All definitions for stage II included the hippocampus, with 3 also comprising the entorhinal cortex (17*,*^25^*,*^26^).

Ten of 16 studies identified stage III as a composite of amygdala, parahippocampal gyrus, fusiform gyrus, and lingual gyrus (11*,*^17^*,*^18^*,*^20^–^23^*,*^25^*,*^26^*,*^28^). The ROIs characterizing stage IV differed widely among studies, but the most cited ones were the inferior and middle temporal cortices, the insula, and the posterior cingulate. One study using ^18^F-AV1451 (21) included, in stage IV, the caudal and rostral anterior cingulate, a structure indicated to be in stage V by 4 studies using ^18^F-MK6240 (17*,*^22^*,*^25^*,*^26^).

The neocortical areas selected to define stage V varied considerably. Still, the definitions captured the notion that tau accumulation is extensive across the neocortex, with the exception of the primary motor and sensory areas. For stage VI, the topographic definition was relatively similar among studies, with the presence of the paracentral, postcentral, and precentral gyri highlighted in most descriptions (11*,*^17^*,*^18^*,*^20^*,*^22^*,*^28^). Divergence was found in the allocation of the cuneus: studies using second-generation ligands associated this structure with stage V (17*,*^22^*,*^25^*,*^26^), and those using first-generation ligands associated it with stage VI (11*,*^18^*,*^20^*,*^21^*,*^23^*,*^28^).

Some variability was also observed in the definitions of meta-ROIs based on the Braak simplified scheme (Supplemental Table 2). Two studies (28*,*^29^) defined Braak I/II as the entorhinal cortex, whereas 2 others (30*,*^31^) included the hippocampus along with the entorhinal cortex. The choice of regions for Braak III/IV and V/VI meta-ROIs is aligned with the fact that they represent, respectively, the limbic and neocortical stages of NFT accumulation. However, the insula, the posterior cingulate, and the lingual gyrus were linked to stage V/VI in one study (29) and to stage III/IV in others (30–32). A discrepancy was again observed regarding the anterior cingulate, which belonged to stage III/IV in one study (30) and to V/VI in another (29).

**FIGURE 3.**
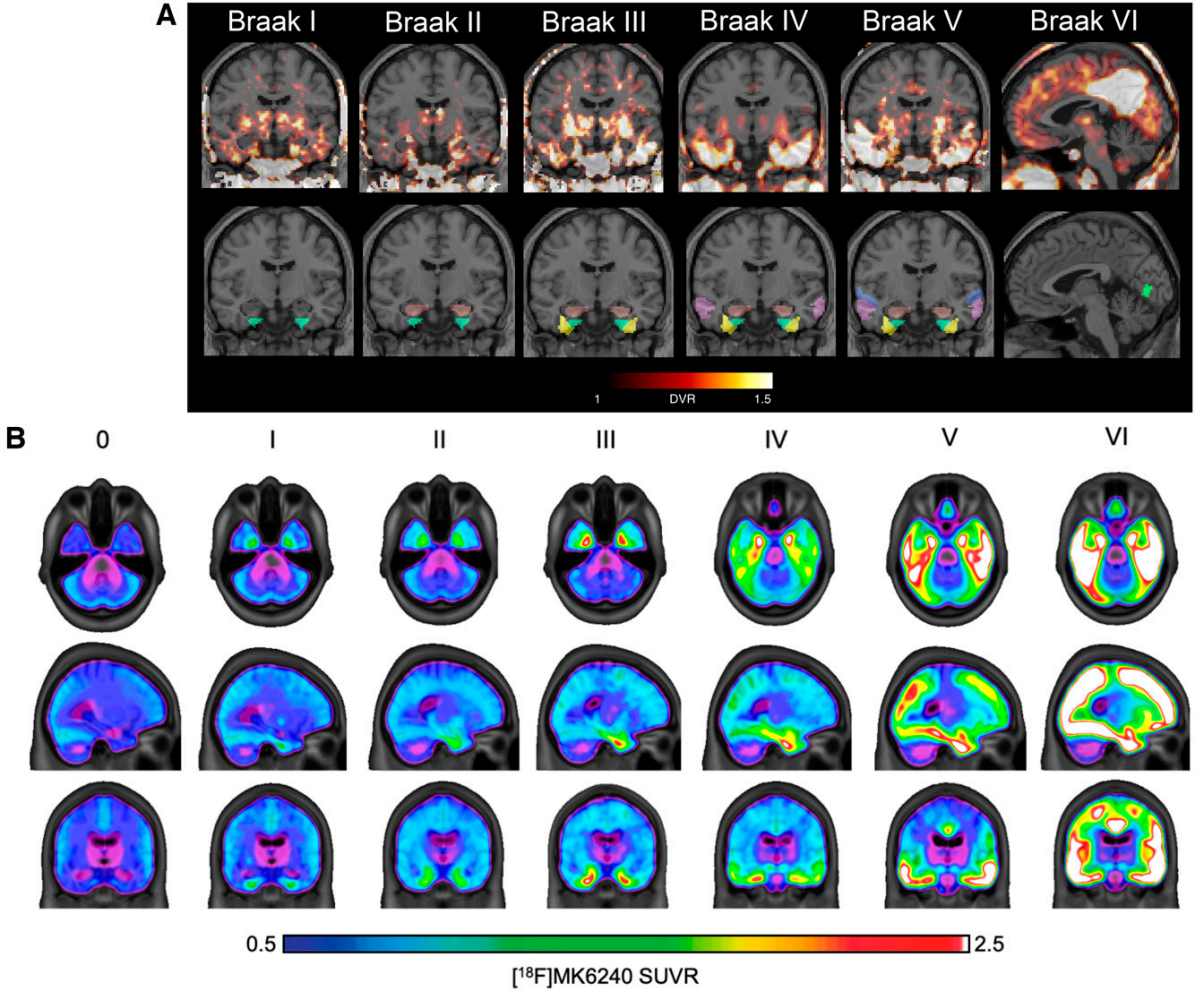
Examples of application of Braak staging in PET imaging studies. (A) Cases representative of each PET-based Braak stage included in Rullman et al. (27). Left column includes parametric ^18^F-PI2620 PET images merged with standard MRI, whereas right column shows MRI images with atlas regions corresponding to each Braak stage (27). (B) Average ^18^F-MK6240 SUV ratios across brains of participants in each PET-based Braak stage group included in Therriault et al. (26). (Adapted with permission of (*26,^27^*).)

Figure 3 provides examples of imaging representation of PET-based Braak stages used in studies included in this review.

### Variability in Methodologic Approaches to PET-Based Braak Staging

Different methods to evaluate PET-based Braak stages were observed (Supplemental Table 3). Most studies used more than one statistical or practical approach, the most common being to treat the measures of ligand uptake as continuous variables (11*,*^17^–^19^*,*^21^–^33^). Thresholds to define abnormal tau accumulation in Braak ROIs were also largely used (11*,*^17^*,*^18^*,*^22^*,*^24^–^27^*,*^29^*,*^32^*,*^34^). Seven studies assigned an individual-level PET-based Braak stage to participants, which usually followed a hierarchic pattern (i.e., individuals with abnormality in later stages also presented abnormalities in earlier stages) (11*,*^17^*,*^18^*,*^24^*,*^25^*,*^26^*,*^27^). However, this condition was not reported in 2 studies (11*,*^18^) and was not achieved in a small percentage of participants in other studies, whose Braak nonconformant status was disclaimed (17*,*^24^*,*^26^*,*^27^). Other approaches included identifying peaks of tau signal (28) or applying gaussian mixture modeling to assess the tau positivity probability (20) in Braak ROIs.

### Correspondence of Postmortem Tau Autoradiography Binding to Braak Histopathologic Staging

In an autoradiographic study including 22 brains, ^18^F-AV1451 binding correlated with the NFT accumulation pattern, demonstrating a promising ability for in vivo estimation of Braak staging (35). Another study found that ^18^F-AV1451 binding in subjects with atypical AD did not entirely follow the early-stage progression as in the Braak scheme, showing moderate correspondence to tau accumulation in cases at stages 0–VI (36).

### Recapitulation of Histopathologic Staging by PET-Based Braak Staging

Most studies indicated that the PET-based Braak staging framework corresponds well with the Braak histopathologic descriptions. Both cross-sectional and longitudinal studies showed that the binding patterns strongly resemble the Braak histopathologic staging (11*,*^17^–^22^*,*^24^–^28^*,*^32^–^35^), even though atypical patterns of NFT accumulation were also observed (17*,*^19^*,*^20^*,*^24^*,*^26^–^28^*,*^34^*,*^36^). Notably, similarities in NFT accumulation patterns were found in postmortem neuropathology and antemortem ^18^F-MK6240 PET data from 2 AD patients who died 12 and 22 mo after the imaging acquisition (25).

Nonetheless, Franzmeier et al. suggested that tau accumulation and epicenters can vary spatially and that tau deposition begins locally and disseminates subsequently throughout functionally connected brain regions (20). Therefore, tau spreading may not always follow the Braak scheme and may be tracked at an individual level according to the normative connectivity patterns of tau epicenters (20). Seemiller et al. used ^18^F-AV1451 PET and resting-state functional MRI to research the relationship between Braak staging and the network degeneration hypothesis, according to which AD degenerative changes affect large brain networks and display functional connectivity patterns (28*,*^37^–^39^). Their results indicate that the connectivity of individual regions displaying tau pathology could predict its progression into higher Braak stages and an increase of tau deposition in earlier Braak stages (28). Thus, these findings agree with the Braak histopathologic staging proposal and corroborate the network degeneration hypothesis (28).

In a cross-sectional study, tau deposition in cognitively unimpaired (CU) individuals observed on ^18^F-AV1451 PET resembled but was not identical to Braak and Braak’s descriptions of early NFT distribution (34). Both elevated tau PET signal in Braak III–VI ROIs and widespread early tau pathology were observed in CU individuals (34). Differences in NFT accumulation were also seen between younger-onset and older-onset AD, with the former presenting higher tau signal in frontal regions (34). This provides evidence of possible variability in NFT deposition, which may be associated with disease phenotypes (34).

### Association of PET-Based Braak Staging with Clinical Measures and Diagnoses

Schöll et al. reported cross-sectional and longitudinal associations between ^18^F-AV1451 uptake and cognitive decline in Braak ROIs (11). These findings were subsequently replicated in other cross-sectional studies, with PET-based Braak stages correlating not only with cognitive impairment but also with global disease severity and clinical diagnosis (17*,*^18^*,*^22^*,*^23^*,*^24^*,*^27^). Therriault et al., in turn, used individual-level PET-based Braak staging and observed that cognitive decline started at stages II–IV and progressed with the advance of Braak stages (26). Specifically, whereas early Braak stages featured isolated memory deficits, late stages had poorer clinical dementia ratings (26).

Moreover, Cho et al. demonstrated that a higher tau accumulation rate in stages I–II was observed in mild cognitive impairment (MCI) patients, followed, respectively, by AD dementia and CU individuals (33). Tau accumulation reached higher rates in Braak III–IV ROIs in the AD dementia group. Moreover, the progression in tau accumulation in areas of higher Braak stage was associated with cognitive decline in AD (33). In another study, individuals classified as Aβ-negative CU, Aβ-positive CU, Aβ-positive MCI, and Aβ-positive AD showed a trend toward longitudinal tau accumulation predominantly in Braak I–II, I–III, IV–V, and V–VI ROIs, respectively (25). In Nihashi et al., ^18^F-THK5351 binding in Braak ROIs did not differ between Aβ-positive and Aβ-negative CU individuals (23). MCI and AD individuals had higher binding than CU participants in Braak I–IV ROIs and in all Braak ROIs, respectively (23). No significant difference was found between the MCI and AD groups. Finally, a cross-sectional study found that ^18^F-RO948 uptake was greater in individuals with AD dementia in all Braak ROIs than in Aβ-positive MCI and Aβ-negative CU patients (29). The Aβ-positive MCI group also presented a higher retention rate in I–II, III–IV, and I–IV ROIs than did Aβ-negative CU participants (29).

### Correspondence of In Vivo PET-Based Braak Staging with Cerebrospinal Fluid (CSF), Plasma, and Neuroimaging Biomarkers of AD

The association of Aβ positivity or load with PET-based Braak staging was pointed out in several studies (11*,*^17^–^19^*,*^22^–^24^*,*^26^). Correlations were also shown with several CSF biomarkers (22*,*^26^), plasma phosphorylated tau (26), and decreased hippocampal volume (11*,*^17^*,*^18^*,*^23^).

Among MCI or AD patients, ^18^F-AV1451 uptake in the entorhinal cortex was linked to local cortical atrophy and, in Braak III–IV and V–VI ROIs, to atrophy in local and distant locations (31). In CU individuals, tau had no effect on gray matter density (31). These findings suggest that tau spatial deposition is closely associated with neurodegeneration in symptomatic individuals in the AD continuum (31).

### Comparisons of PET-Based Braak Staging and Other Strategies to Stage Individuals in the AD Continuum

Braak ROI-based staging demonstrated performance similar to that of whole-brain and regional tau measures in differentiating individuals with MCI and AD from Aβ-negative CU individuals. Nonetheless, PET-based Braak staging showed an enhanced potential to determine the initial stages of tau deposition (18). Furthermore, Braak ROI SUV ratio outperformed MRI and CSF measures when distinguishing AD and MCI from CU and non-AD groups (29).

In Kim et al., ^18^F-THK5351 PET was compared with MRI brain volumetry of selected regions (cingulate isthmus, inferior parietal lobule, and hippocampus) and with the combination of these tools regarding their ability to discriminate AD from CU and MCI (30). The combined model presented better performance than the SUV ratio of Braak ROIs alone in distinguishing AD from CU but not AD from MCI. Comparable performance was found for the combined model and MRI brain volumetry. In sum, all methods demonstrated good performance in distinguishing the phases of the AD continuum, with an additional value of brain volumetry in the distinction between AD and CU (30).

## DISCUSSION

With this review, we aimed to examine the literature about the use of tau PET to stage AD using ROIs and meta-ROIs that resemble the Braak histopathologic classification. The Braak histopathologic framework shows a good correspondence with clinical status across the AD spectrum (40–42), and tau PET is a promising tool to make possible in vivo AD staging using this classification. Despite the limited literature on the topic, most of the available studies indicate that tau PET performs well in staging AD when using Braak ROIs or meta-ROIs, even when compared with other biomarkers and clinical predictors.

The adaptation of the original histopathologic descriptions of Braak stages to their use in tau PET imaging studies has been done taking into account the limitations of PET imaging. This is evidenced by the variability in the anatomic definitions of Braak ROIs and meta-ROIs across studies, which may result from factors such as variability in imaging acquisition technique, tracers used, and the aims of the study. Also, it should be noted that the Braak histopathologic scheme was revised after the improvement of histopathology techniques. Some minor discrepancies in the topographic definitions of the stages are observed between different reports by Braak et al. (6*,*^7^*,*^43^–^45^). Insula involvement, for instance, was associated with stage V in the original description (6) but with stage IV in more recent studies (7*,*^44^*,*^45^). Changes in the description of the progression of neocortical damage are also seen between the initial and subsequent studies (6*,*^7^*,*^43^–^45^).

We observed multiple definitions of Braak I as the entorhinal cortex, which was described by Braak and Braak to be uninvolved or minimally involved at this stage (6*,*^7^). Partial anatomic descriptions were observed for stage II, whose hallmarks are the entorhinal cortex and hippocampal sector CA1 involvement (6*,*^7^). The limited spatial resolution of PET may partially account for these discrepancies since it hampers tau pathology identification in small areas of the medial temporal lobe and signal discrimination in the transentorhinal and entorhinal cortices. Braak I–II ROI definitions in ^18^F-AV1451 PET studies may be also affected by off-target binding to the choroid plexus, which limits the evaluation of early Braak ROIs (12). Even though some incompleteness was seen in stage III descriptions, the choice of ROIs had a high interstudy agreement and adequate correspondence with postmortem observations (6*,*^7^). The ROIs representing stages IV and V varied considerably but were in line with the fact that NFT pathology progresses more broadly into the associative neocortex in stage IV and extends to nearly all remaining neocortical areas (sparing primary fields) in stage V (6*,*^7^). For stage VI, definitions were fairly similar among studies and agreed with the histopathology framework (6*,*^7^). The definition of meta-ROIs also showed discrepancies, with incompleteness for Braak I/II and inclusion of regions linked mainly to stages III/IV (the insula, the posterior cingulate, and the lingual gyrus) in the V/VI meta-ROI (6*,*^7^). The relevance of these inconsistencies constitutes a knowledge gap and might be affected by the size of those regions relative to the Braak ROIs and by the resolution of tau PET imaging. Since the Braak framework serves as a benchmark for AD progression, the standardization of Braak ROIs might be important to test its accuracy as a staging and prognostic tool and to allow reproducibility.

Regardless of those discrepancies, most studies demonstrated that the tau accumulation seen in PET seems to follow the Braak histopathologic framework (11*,*^17^–^22^*,*^24^–^28^*,*^32^–^35^). Different clinical, fluid, and imaging biomarkers of AD severity were found to be associated with progressive tau deposition in subsequent Braak stages or a stronger signal in Braak ROIs: cognitive decline (11*,*^17^*,*^22^–^24^*,*^26^*,*^27^*,*^33^), dementia severity (17*,*^26^), amyloid positivity or load (11*,*^17^–^19^*,*^22^–^24^*,*^26^), CSF measures (22*,*^26^), plasma phosphorylated tau (26), diminished hippocampal volume (11*,*^17^*,*^18^*,*^23^), and cortical atrophy (31). Furthermore, PET-based Braak staging appears to present a comparable or superior performance in relation to other AD staging methods (18*,*^29^*,*^30^). These findings reinforce the potential use of the proposed staging scheme in living individuals. Nonetheless, further studies comparing PET-based Braak staging to other methods assessing AD severity (e.g., fluid biomarkers or tau PET signal quantification in specific ROIs) are needed, especially concerning cost-effectiveness.

PET-based Braak staging also performed well in identifying the preclinical phases of AD. This ability is relevant in the context of population enrichment strategies for disease-modifying clinical trials. Indeed, this clinically silent period has become an attractive stage for preventive therapeutic interventions (46). Apart from that, this staging strategy could be useful for earlier AD diagnosis, before symptom onset, and for the proposition of personalized care strategies (47).

The existence of patterns of tau deposition other than the one proposed by Braak and Braak has been highlighted in several studies (17*,*^19^*,*^20^*,*^24^*,*^26^–^28^*,*^34^*,*^36^). One of these studies (20) proposes links between Braak-nonconformant patterns and tau spreading based on brain connectivity patterns. Furthermore, Vogel et al. proposed, via network diffusion models, the occurrence of different spatiotemporal patterns of tau accumulation in AD: limbic-predominant, medial temporal lobe–sparing, and posterior and lateral resembling atypical AD phenotypes (48). These data-driven techniques refine the concept of hierarchic patterns of tau propagation by emphasizing the role of brain connectivity in shaping their sequence. Moreover, studies on atypical AD have led to the emerging perspective that some individuals, because of their atypical NFT distribution, do not fit the Braak staging framework. Here, it is important to consider what a lack of fit to the Braak staging framework means. For example, do individuals with a significantly greater frontal tau load than typically observed go against the Braak staging system? What if the medial temporal lobe is concurrently affected, albeit to a lesser degree? Although tau PET studies have indicated that the regional distribution of tau pathology at the individual level is indeed highly variable, it is presently unclear to what extent different clinical variants of AD have tau patterns that go against the Braak staging framework.

Some limitations were identified in the available literature on PET-based Braak staging. First, Braak histopathologic staging relies on the use of staining techniques to detect specific tau phosphorylation sites (49), but the extent of their contribution to the tau PET signal is yet to be clarified. Moreover, the fact that there is no established framework for defining Braak-based ROIs or meta-ROIs may lead to the misrepresentation of Braak histopathologic stages in imaging studies. Similarly, there is no standardization regarding the methods to establish thresholds for Braak ROI positivity. This includes the choice of the reference control group, which varied especially with respect to age across studies. Furthermore, the most used ligand in the available studies is ^18^F-AV1451, whose off-target binding may compromise the assessment of early Braak ROIs (12). Other ligands are still underrepresented in the literature, and there is a need for more studies testing and comparing their staging properties. Further studies comparing PET-based Braak staging with other biomarkers of disease severity are also necessary to understand the real potential of this staging method. Here, we suggest, for example, evaluating both its individual and combined efficiencies with other CSF, plasma, and imaging biomarkers. Also, most studies are not clear regarding which clinical variants of AD are evaluated. Studies addressing different patterns of tau deposition in early- and late-onset AD and comparing the typical and atypical phenotypes of AD are warranted to increase our understanding of the limitations of PET-based Braak staging.

It is also critical to consider the limitations of neuropathologic Braak staging itself, which was developed more than 30 y ago using techniques insensitive to the detection of NFT heterogeneity across various brain regions (6). Recent neuropathologic studies refined the initial contributions of Braak and Braak by identifying subtypes of AD with alternative distributions of NFTs at autopsy (50). Since it is based on autopsy assessments, another caveat of Braak and Braak’s description is due to the cross-sectional nature of pathologic observations, which prevents longitudinal analyses. Furthermore, the relatively small number of cortical areas typically needed for neuropathology staging limits the observation of interindividual differences in tau distribution. Braak neuropathologic staging is also based on semiquantitative measures, making it difficult to classify cases in a transitional state.

## CONCLUSION

Overall, most studies suggest that PET-based Braak staging may be a logical starting point for staging AD, as it showed a valuable ability to discriminate between the phases of the AD continuum and correlated with clinical, laboratory, and imaging biomarkers of disease severity. Refinements in this staging system are necessary to incorporate atypical and Braak-nonconformant cases. Further studies are needed to validate tau staging systems in research and clinical practice. They should address, for instance, the correspondence with other clinical (e.g., performance in activities of daily living) and biologic markers of AD, include comparisons between in vivo PET and postmortem neuropathologic observations, and check the progression of PET-based Braak stages at an individual level, preferably for longer follow-up periods. Also, the harmonization of Braak ROIs and methods to define thresholds for tau abnormality might represent a further step for ascertaining reproducibility across studies.

## DISCLOSURE

Serge Gauthier has served as a scientific advisor to Cerveau Therapeutics. Eduardo Zimmer serves on the scientific advisory board of Next Innovative Therapeutics. No other potential conflict of interest relevant to this article was reported.
